# An Updated View on the Rck Invasin of *Salmonella*: Still Much to Discover

**DOI:** 10.3389/fcimb.2017.00500

**Published:** 2017-12-08

**Authors:** Julien Mambu, Isabelle Virlogeux-Payant, Sébastien Holbert, Olivier Grépinet, Philippe Velge, Agnès Wiedemann

**Affiliations:** ^1^Institut National de la Recherche Agronomique, UMR1282 Infectiologie et Santé Publique, Nouzilly, France; ^2^Université François Rabelais, UMR1282 Infectiologie et Santé Publique, Tours, France

**Keywords:** *Salmonella*, gene expression, outer membrane protein, membrane receptor, bacterial internalization, cell signaling, bacterial behavior, organ colonization

## Abstract

*Salmonella* is a facultative intracellular Gram-negative bacterium, responsible for a wide range of food- and water-borne diseases ranging from gastroenteritis to typhoid fever depending on hosts and serotypes. *Salmonella* thus represents a major threat to public health. A key step in *Salmonella* pathogenesis is the invasion of phagocytic and non-phagocytic host cells. To trigger its own internalization into non-phagocytic cells, *Salmonella* has developed different mechanisms, involving several invasion factors. For decades, it was accepted that *Salmonella* could only enter cells through a type three secretion system, called T3SS-1. Recent research has shown that this bacterium expresses outer membrane proteins, such as the Rck protein, which is able to induce *Salmonella* entry mechanism. Rck mimics natural host cell ligands and triggers engulfment of the bacterium by interacting with the epidermal growth factor receptor. *Salmonella* is thus able to use multiple entry pathways during the *Salmonella* infection process. However, it is unclear how and when *Salmonella* exploits the T3SS-1 and Rck entry mechanisms. As a series of reviews have focused on the T3SS-1, this review aims to describe the current knowledge and the limitations of our understanding of the Rck outer membrane protein. The regulatory cascade which controls Rck expression and the molecular mechanisms underlying Rck-mediated invasion into cells are summarized. The potential role of Rck-mediated invasion in *Salmonella* pathogenesis and the intracellular behavior of the bacteria following a *Salmonella* Rck-dependent entry are discussed.

## Introduction

*Salmonella* is a Gram-negative, facultative anaerobic and gastrointestinal pathogen belonging to the *Enterobacteriaceae* family. Within the genus *Salmonella*, two species, *Salmonella bongori* and *Salmonella enterica* have been identified. *S. enterica* is divided into six sub-species based on their biochemical and genomic properties: *enterica, salamae, arizonae, diarizonae, houtenae*, and *indica* (Tindall et al., 2005). To date, the *S. enterica* species includes more than 2,600 serotypes and most of them are able to infect a wide spectrum of plant and animal hosts including humans. *Salmonella* infection causes salmonellosis, which is a water- or food-borne disease, ranging from gastroenteritis to typhoid and paratyphoid fever according to hosts and serotypes. In humans, gastroenteritis is caused by serotypes with a broad spectrum of hosts, such as *S*. Typhimurium and *S*. Enteritidis, whereas typhoid and paratyphoid fever are induced by host-restricted serotypes, *S*. Typhi and *S*. Paratyphi, respectively (Velge et al., 2005; Bäumler and Fang, 2013).

Following ingestion, *Salmonella* survives gastric acidity to reach the intestine. Within the intestinal lumen *Salmonella* exploits inflammation to take advantage over the intestinal microbiota (Stecher et al., 2007). A crucial step for *Salmonella* infection to establish is the passage through the intestinal barrier. To achieve this, *Salmonella* can rely on its ability to induce its own entry into enterocytes and M cells, or it can be taken in through direct capture by CD18-expressing phagocytes (Watson and Holden, 2010). *Salmonella* colonization is restricted to the host intestine during gastroenteritis. However, *Salmonella* can disseminate to extra-intestinal sites through the blood, leading to the colonization of deep organs in systemic diseases via its invasion into different cell types including hepatocytes in the liver (Lin et al., 1987; Conlan and North, 1992).

To penetrate into non-phagocytic cells, *Salmonella* has developed different mechanisms which involve hijacking host cell signaling and processes (Velge et al., 2012; LaRock et al., 2015). The first and most studied invasion factor of *Salmonella* is a type three secretion system, called T3SS-1 encoded by the *Salmonella* pathogenicity island-1 (SPI-1). This secretion system allows the translocation of *Salmonella* effectors directly into the host cell leading to bacterial internalization (Galán and Collmer, 1999). Until recently, it was thought that *Salmonella* only invaded cells using the T3SS-1, promoting a trigger entry mechanism mainly characterized by massive rearrangements of the cell surface and actin cytoskeleton at the entry site (Cossart and Sansonetti, 2004). However, to initiate contact with and entry into host polarized cells in a T3SS-1 dependent way, *Salmonella* seems to require a non-fimbrial giant adhesin SiiE, encoded by SPI-4 (Barlag and Hensel, 2015). Recent data have shown that, even in the absence of the T3SS-1, *Salmonella* remains able to invade epithelial and fibroblastic cell lines and to enter into 3-D intestinal epithelial cells (Aiastui et al., 2010; Radtke et al., 2010; Rosselin et al., 2011), indicating the existence of other invasion factors. These results are consistent with the fact that a *Salmonella* strain lacking SPI-1 was isolated from a human food-borne disease outbreak in China (Hu et al., 2008). In addition, it has been shown that *S*. Typhimurium lacking T3SS-1 causes an enteric disease in murine and bovine infection models (Coombes et al., 2005) and *S*. Enteritidis lacking T3SS-1 generates a systemic disease in chicken (Desin et al., 2009). Several studies have identified invasins expressed by *Salmonella* and have clearly established that *Salmonella* can adhere and invade cells using the outer membrane proteins PagN (PhoP activated gene N) or Rck (resistance to complement killing) (Lambert and Smith, 2008; Rosselin et al., 2010). Moreover, a mutant *Salmonella* strain, which does not express T3SS-1, Rck or PagN, is still able to invade several epithelial cell lines, demonstrating that unknown invasion factors remain to be identified (Rosselin et al., 2011). Thus, *Salmonella* is able to use multiple entry pathways. However, the question remains as to how and when *Salmonella* exploits these different entry pathways and to whether they operate in parallel or at different relevant steps in the infection process.

In this review, we will focus only on our current knowledge of the Rck outer membrane protein, the most characterized invasin of *Salmonella*. We will present its virulence-associated properties and how its expression is regulated. The entry mechanism following its direct interaction with the host cell surface and its role in *Salmonella* pathogenesis will also be reviewed. Finally, we will discuss the intracellular lifestyle of *Salmonella* following Rck-mediated entry. The aspects of *Salmonella* invasion mechanisms, which are not covered in our review, are well described in the review of Hume et al. (2017).

## The Rck protein

The 17.4 kDa Rck protein belongs to the Ail/Lom protein family that consists of several bacterial or phage outer membrane proteins (OMP) involved in the virulence of Gram-negative pathogens expressing these proteins. These OMPs share a common predicted structure, i.e., eight transmembr ane beta-sheets and four cell surface-exposed loops. While the transmembrane segments are well conserved among the members of the Ail/Lom protein family, the loops are not (Cirillo et al., 1996). This could explain the various functions exerted by the members of the family such as resistance to complement, adhesion, invasion, bacterial survival in macrophages or host persistence (Miller et al., 1992; Crago and Koronakis, 1999; Nishio et al., 2005; Atkinson and Williams, 2016). All the known mature Rck proteins are 161 amino-acid long and share an identity of above 98%. An auto-aggregation motif spanning 80-89 amino acids has recently been identified in the second cell surface-exposed loop and is 100% conserved in all Rck proteins. This motif is not present in the other members of the Ail/Lom family (Glaubman et al., 2016). Moreover, the third loop and more precisely the 46 amino-acid region from G114 to V159 is necessary and sufficient to induce the invasion process. This region is very well conserved except for one His
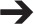
Arg amino-acid substitution at position 125 between the Rck proteins of *S*. Typhimurium and *S*. Enteritidis.

## From the *pefI-srgC* operon to the regulation of Rck expression

**Figure 1:**
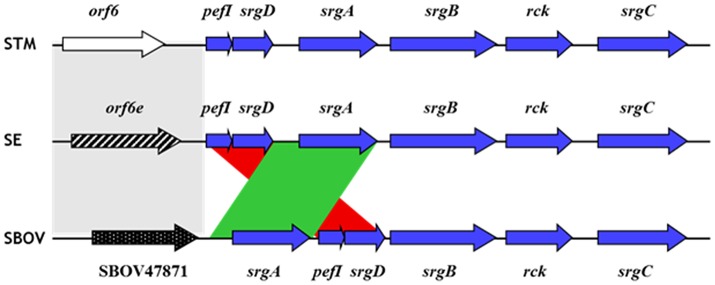
Alignment of the *pefI-srgC* operons and upstream region of *Salmonella*. The six ORFs belonging to the *pefI-srgC* operon are represented by blue arrows. The red and green colored areas indicate the gene rearrangement between SBOV and the two other serotypes. The gray colored area represents heterologous sequences between the three serotypes. STM, *S*. Typhimurium; SE, *S*. Enteritidis; SBOV, *S*. Bovismorbificans.

The Rck protein is encoded by the *rck* open reading frame (ORF) carried on the *pefI-srgC* operon present only on the large virulence plasmid of *Salmonella*. To date, the complete operon, and consequently the *rck* ORF, has only been identified in *S*. Bovismorbificans and in the two most frequently isolated serotypes in humans during gastrointestinal infections, i.e., *S*. Typhimurium and *S*. Enteritidis (Rychlik et al., 2006; Bronowski et al., 2013). In *S*. Typhimurium, this operon contains six ORFs: *pefI, srgD, srgA, srgB, rck*, and *srgC*. The structure of the operon is slightly different in the three serotypes even though the nucleotide sequence is well conserved (Figure 1). In *S*. Enteritidis, the order of the ORFs is the same as in *S*. Typhimurium, but differs in *S*. Bovismorbificans. In the latter serotype, the *srgA* ORF is upstream of *pefI* instead of being positioned after *srgD*, but the structure of the rest of the operon is conserved. Moreover, in *S*. Enteritidis and *S*. Bovismorbificans, the 5′ region upstream of *pefI* that contains the promoters responsible for the transcription of the operon is completely different. As a consequence, in *S*. Enteritidis the transcription and the regulation of the *pefI-srgC* operon has been shown to be different from that in *S*. Typhimurium (see below) (Abed et al., 2014). The distribution of the *pefI-srgC* operon and consequently that of *rck* is probably not restricted to these three serotypes. Indeed, sequences of the virulence plasmid are only available for a few other serotypes. This is the case for *S*. Choleraesuis, Dublin, Gallinarum, Paratyphi C, or Pullorum serotypes (Hong et al., 2008; Liu et al., 2009; Richardson et al., 2011; Feng et al., 2012). The recent identification of the *pefI-srgC* operon on the virulence plasmid of *S*. Bovismorbificans serotype (Bronowski et al., 2013) and the fact that several papers describe the existence of a virulence plasmid and of *srgA* in other serotypes (Popoff et al., 1984; Woodward et al., 1996; Bouwman et al., 2003) support the hypothesis that this operon is more widely distributed among *Salmonella* than initially thought. The current massive sequencing of *Salmonella* genomes will help to clarify how the large virulence plasmid is distributed among the serotypes and thus also the distribution of the *pefI-srgC* operon.

The ORFs of the *pefI-srgC* operon encode proteins that have not yet been phenotypically related. *pefI* encodes a transcriptional regulator involved in the repression of the operon encoding Pef fimbriae and located immediately upstream of the *pefI-srgC* operon. The *srgD* ORF encodes a putative transcriptional regulator. The only result suggesting a role of this sequence is that described by Wozniak et al. and Wallar et al. showing that the *pefI-srgD* locus is involved in the negative regulation of flagellar genes and thus, in the repression of bacterial motility (Wozniak et al., 2009; Wallar et al., 2011). SrgA is required for the post-translational maturation of the PefA protein, the major subunit of the Pef fimbriae, and likewise, of the SpiA protein of the Type III secretion system 2 required for *Salmonella* intracellular survival. SrgA is a disulfide oxydoreductase homologous to DsbA (disulfide bond A) that catalyzes disulfide bond formation and is thus important in the correct folding of these proteins (Bouwman et al., 2003; Miki et al., 2004). The last two ORFs of the operon, *srgB* and *srgC*, encode a putative lipoprotein and a transcriptional regulator of the AraC family respectively. This raises the important question of what is the relationship between the six ORFs encoded by the *pefI-srgC* operon. In general, ORFs that are co-transcribed are phenotypically related. *pefI* and *srgA* are both involved in Pef fimbriae biosynthesis. To date, no link between these ORFs and the two putative transcriptional regulators (SrgD and SrgC), the Rck invasin or the putative lipoprotein SrgB has been found. However, regarding the involvement of PefI and SrgA in Pef fimbriae biosynthesis, it could be hypothesized that the Rck invasin and Pef fimbriae play a role in *Salmonella* pathogenesis in a similar environment and could cooperate. Currently, little is known about the environmental conditions required for Pef and Rck expression. Both are expressed at 37°C but not at 30°C. Pef are known to be expressed in an acidic pH in standing culture conditions, while Rck expression has only been detected in swarming conditions in the presence of acyl-homoserine-lactones (AHLs) (Nicholson and Low, 2000; Kim and Surette, 2006). As Pef fimbriae are involved in biofilm formation, fluid accumulation in the infant mouse and colonization of different animals, i.e., in chicken, pigs, mice, and cattle (Bäumler et al., 1996; Lawley et al., 2006; Ledeboer et al., 2006; Chaudhuri et al., 2013), it would be interesting to study the role of Rck and of the other proteins of the *pefI-srgC* operon in these models.

**Figure 2:**
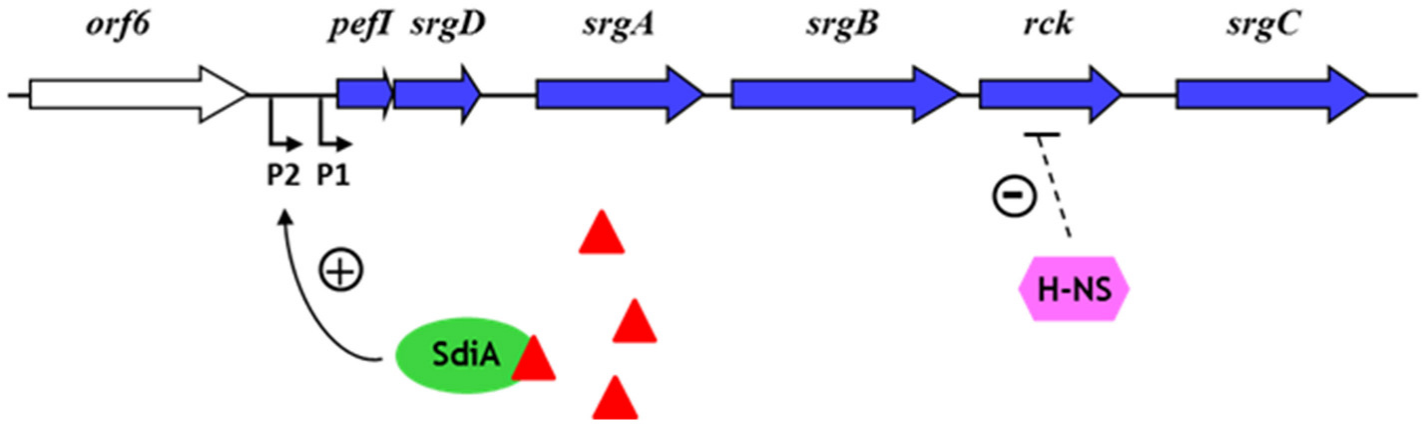
Regulation of *rck* expression in *S*. Typhimurium. The six ORFs belonging to the *pefI-srgC* operon are represented by blue arrows. The two promoters identified upstream of *pefI* are indicated by broken arrows (Abed et al., 2014). Red triangles represent acyl-homoserine lactones (AHLs). The dashed line for negative H-NS regulation means that the target of H-NS is unknown.

The Rck protein is not expressed under common laboratory culture conditions. Two transcriptional regulators have been shown to regulate the transcription of the *pefI-srgC* operon and consequently the expression of Rck in *S*. Typhimurium. The quorum sensing transcriptional regulator SdiA (suppressor of cell division inhibition A) activates the transcription of the *pefI-srgC* operon directly at 37°C but not at 30°C or below. This activation takes place on the most distal promoter identified upstream of *pefI* and requires the addition of AHLs in the culture medium (Figure 2) (Abed et al., 2014). *Salmonella* does not produce AHLs but it can detect AHLs produced by other bacteria. AHL binding induces a folding switch of SdiA, which allows SdiA to interact efficiently with its target DNA region (Yao et al., 2006). This regulation through quorum sensing and an expression at 37°C but not below supports a role of Rck and the other proteins of the operon in the intestine of warm-blooded animals. However, bacteria colonizing the intestine of healthy mammals seem not to synthesize AHLs or only in quantities insufficient to activate the SdiA regulon suggesting that other positive regulatory mechanisms remain to be found (Erickson et al., 2002; Smith et al., 2008). It is noteworthy that the transcriptional regulation of the *pefI-srgC* operon differs between *S*. Typhimurium and *S*. Enteritidis due to their different nucleotide sequence present in the 5′ region upstream of this operon. SdiA regulates Rck expression in *S*. Enteritidis but at a lower level than in *S*. Typhimurium and in an AHL-independent manner (Abed et al., 2014). The second regulator of the *pefI-srgC* operon known to date is the nucleoid-associated protein H-NS that strongly silences Rck expression at both 30 and 37°C. H-NS is involved in the negative regulation of many bacterial genes including virulence genes in pathogens (Figure 2). The mechanism of Rck regulation by H-NS is currently under investigation in our laboratory, as neither of the two promoters upstream of the *pefI-srgC* operon seems to be targeted by H-NS (Abed et al., 2014). Characterizing the regulation mechanisms of the *pefI-srgC* operon further will help to understand the precise role of Rck in *Salmonella* pathogenesis.

## From the receptor to a zipper-like entry mechanism

**Figure 3:**
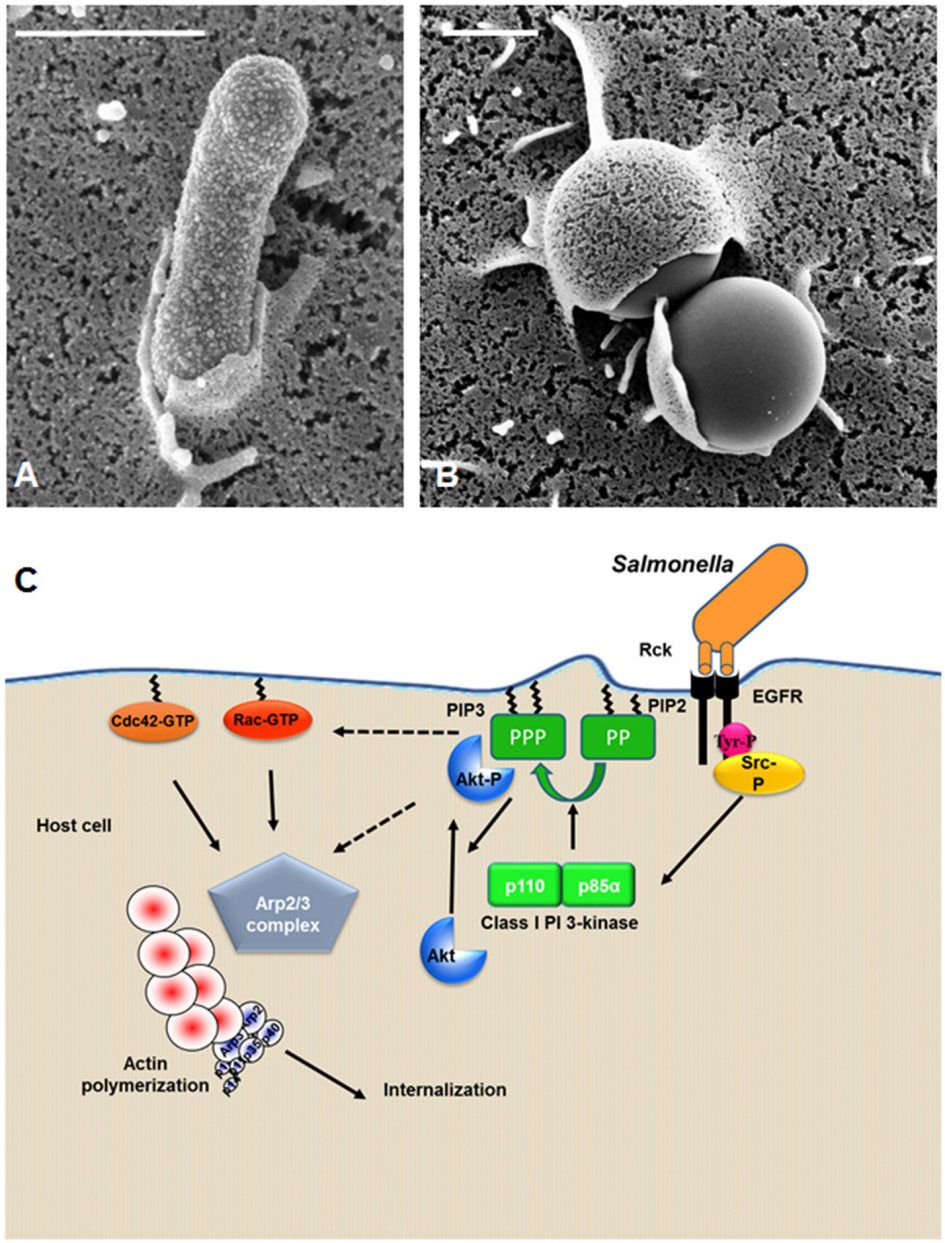
Mechanism of *Salmonella* entry mediated by Rck invasin. **(A,B)** Scanning electron microscopy of zipper mechanism induced by Rck. Human choriocarcinoma (Jeg-3) cells infected with **(A)** *E. coli* overexpressing Rck; **(B)** Rck-coated beads. **(A,B)** Bar = 1 μm. **(C)** A model of Rck-mediated intracellular signaling cascade leading to *Salmonella* internalization. The interaction of Rck with the EGFR induces a cellular signaling pathway, leading to internalization. The signaling cascade requires different cellular proteins such as c-Src kinase, PI3-kinase, Akt, Cdc42 and Rac GTPases, the Arp2/3 complex and actin. Dotted arrows: possible interactions and/or signaling events.

*Salmonella* is able to induce eukaryotic cell invasion by using Rck invasin, which binds to the host plasma membrane. Research has shown that when *Salmonella* is grown in the presence of AHLs under swarming conditions, known to induce SdiA expression, a *rck* mutant strain of *Salmonella* has impaired cell invasion compared to the wild-type strain (Rosselin et al., 2010). In addition, when Rck is overexpressed in a non-invasive *E. coli* strain, or coated on latex beads, it is sufficient to promote cell invasion by a receptor-dependent process called a zipper mechanism (Heffernan et al., 1994; Rosselin et al., 2010). More precisely, a minimal region containing 46 amino acids (G114 to V159) has been identified as necessary and sufficient to promote this mechanism. Further analyses using scanning and transmission electron microscopy have shown different steps of entry including microvillus-like extensions and weak membrane rearrangements, as observed in Figures 3A,B. These results are similar to those observed during *Listeria monocytogenes* invasion (Cossart and Sansonetti, 2004). Therefore, *Salmonella* is able to invade eukaryotic cells using a zipper mechanism, making *Salmonella* the first bacteria ever identified to penetrate the host cell by both a trigger and a zipper mechanism. Unlike a trigger mechanism, a zipper entry mechanism is observed after a ligand protein expressed at the bacterium surface interacts with a host cell receptor. This interaction leads to weak cytoskeleton rearrangements and membrane extensions (Cossart and Sansonetti, 2004). A recent study carried out using Rck -expressed *E. coli* and -coated latex beads has identified the epidermal growth factor receptor (EGFR) *as* the receptor for Rck (Wiedemann et al., 2016). In this study, we demonstrated that varying EGFR expression on the cell surface altered *Salmonella* internalization dependent on Rck. Rck was also shown to interact directly with the extracellular domain of EGFR, leading to internalization of the complex. The domain of Rck interacting with EGFR seems distinct from that of EGF, as EGF competition experiments in the presence of EGF did not interfere with Rck-mediated invasion. The amino acids in the extracellular domain of EGFR necessary to activate the signaling cascade and leading to Rck-mediated internalization remain to be identified. EGFR is a receptor tyrosine kinase, whose binding of ligand allows homo- or hetero-dimerization with a member of the EGFR family and activation of the receptor (Roskoski, 2014). Further studies are required to identify the homo- and hetero-dimer, including EGFR, necessary to induce Rck-mediated invasion.

The first step in activating the signaling cascade induced by the activation of the EGFR is the autophosphorylation of specific tyrosines located in the EGFR cytosolic tail. For example, phosphorylation of the tyrosine located in position 1173 in the cytosolic domain of EGFR is required for Rck-mediated internalization (Wiedemann et al., 2016). The literature clearly describes that phosphorylation of specific tyrosine residues leads to the recruitment and activation of signaling proteins such as the c-Src kinase family through its SH2 (Src Homology 2) domain interacting with phosphotyrosine residues (Furcht et al., 2015). A role of c-Src has been demonstrated during the Rck-mediated signaling cascade leading to internalization. Indeed, c-Src is activated early in the Rck-mediated internalization process and the pharmacological Src family inhibitor prevents bacterial entry. Moreover, c-Src inhibition prevents the PI3-kinase activation, and more particularly that of the p85α-p110 heterodimer at the entry site (Mijouin et al., 2012; Wiedemann et al., 2012). c-Src is recruited and activated at the cytosolic tail of EGFR, where it interacts with the p85 subunit of the PI3-kinase. PI 3-kinase is a lipid kinase crucial in many signaling cascades. This enzyme produces phosphoinositides locally by catalyzing the phosphorylation of PI(4,5)P_2_ in PI(3,4,5)P_3_ at the cell plasma membrane, which promotes recruitment of further cellular effectors such as the prosurvival kinase Akt. Akt is a serine/threonine kinase, which once activated, plays a role within the inner leaflet of the plasma membrane. In the Rck-invasion pathway, Akt activation occurring in the cell membrane via the phosphorylation at Ser473 is required and is probably involved in recruiting other actin-remodeling proteins needed for *Salmonella* invasion by binding to PI(4,5)P_3_. Several guanine nucleotide exchange factors (GEFs) of the small Rho GTP-binding protein family possess PH (pleckstrin homology) domains, enabling interaction with PI(3,4,5)P_3_ and favoring the Rho GTPases translocation to the plasma membrane. The literature describes a complex relationship between PI3-kinase and Akt activation and Rho family GTPases as the latter seems to be able to act both downstream and upstream of PI3-kinase and Akt (Welch et al., 2003; Yang et al., 2012). The Rck-mediated signaling cascade requires the small Rho GTPase family members, Rac1 and Cdc42. The recruitment of Rac1 at the entry site is well documented (Rosselin et al., 2010; Mijouin et al., 2012). However, the process required for Rac1 activation remains unclear. The recruitment and activation of the small Rho GTPase family members impact the actin dynamics of the host cell. More precisely, actin polymerization and nucleation, which leads to actin reorganizing locally, occurs at the entry site and is mediated by the recruitment and activation of actin nucleating proteins, constituting the Arp2/3 complex (Figure 3C). The final step of the Rck-mediated internalization is the formation of a weak membrane rearrangement permitting the wrapping of the bacteria (Rosselin et al., 2010). Further research is needed to understand how the interaction of Rck with the EGFR modulates the membrane rearrangement and fusion, resulting in a vacuolar compartment forming during entry.

## Consequences of Rck-dependent entry on *Salmonella* behavior

Following host cell invasion, *Salmonella* has to survive and proliferate within eukaryotic cells. For that, *Salmonella* resides and replicates either freely within host cell cytosol or within a membrane-bound vacuole called a *Salmonella*-containing vacuole (SCV) (Knodler, 2015). However, the reason why *Salmonella* has developed these alternative processes is not clearly understood and the consequences of the type of initial entry mechanism used on the subsequent *Salmonella* intracellular lifestyle remains to be determined.

Our knowledge to date regarding cell invasion and intracellular survival in a SCV is summarized below. To invade non-phagocytic cells *Salmonella* has developed different mechanisms. The mechanism, which has been elucidated the most, requires T3SS-1 to translocate several bacterial effectors. These effectors interact with host cell partners, inducing profound cytoskeleton and membrane rearrangements, which lead to the bacterium being internalized in an early SCV (30 min following internalization), which then matures to an intermediate state by acquiring macropinocytic and endocytic components (between 30 min and 4 h following internalization). Following this there are several possibilities: (i) xenophagy in epithelial cells allowing cytosolic pathogen destruction (Noad et al., 2017); (ii) SCV lysis governed by up-regulation of T3SS-1 effectors, allowing hyper replication of cytosolic bacteria (Knodler et al., 2014); (iii) acquisition of late endosomal and lysosomal markers, and acidification of luminal pH, characterizing the SCV maturation (Steele-Mortimer, 2008). This environment contributes to T3SS-2 effector expression, which is required for *Salmonella*-induced filament (SIF) formation and intravacuolar replication (Malik-Kale et al., 2011). SCV biogenesis is dependent on T3SS-1 and T3SS-2 effectors, which in a simplistic scheme are sequentially expressed. In fact, some T3SS-1 effectors persist and balance T3SS-2 effector action in the late SCV stages (Brawn et al., 2007). Moreover, T3SS-1 effector delivery persists over time during *Salmonella* cytosolic replication (Finn et al., 2017).

**Figure 4:**
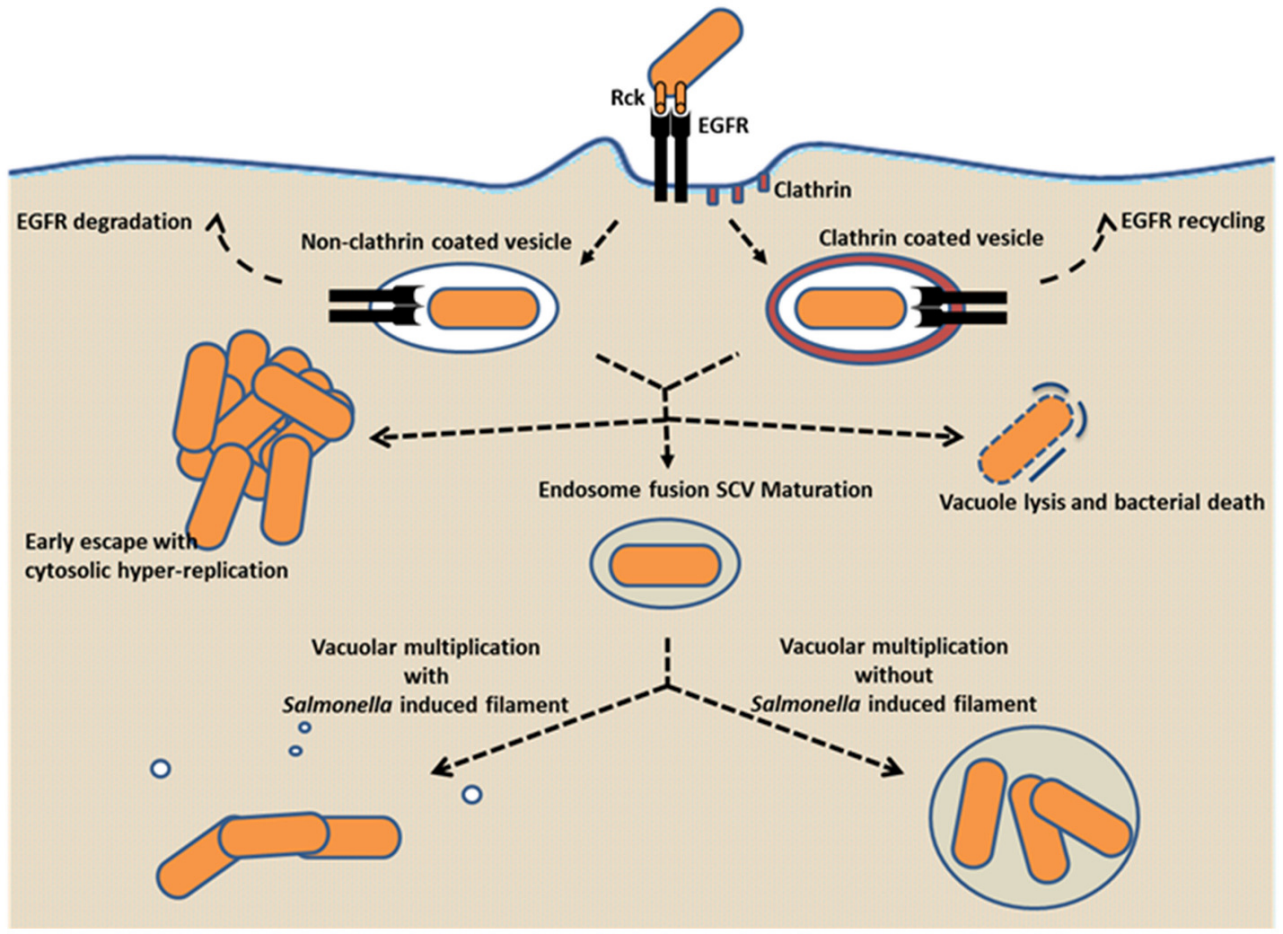
Potential intracellular behaviors of *Salmonella* following Rck-mediated invasion. Rck interaction with EGFR could lead to internalization of *Salmonella* in non-clathrin or clathrin-coated vesicule. Then vacuole escape or vacuole lysis could lead to *Salmonella* cytosolic hyper-replication or bacterial death respectively. As commonly described, expression of T3SS-1 effectors relayed by expression of T3SS-2 effectors allows vacuole maturation leading to *Salmonella* multiplication in vacuole with or without SIF formation. Dotted arrows represent the potential outcomes.

The fact that we now know that *Salmonella* can enter cells through Rck, interacting with EGFR raises the question as to how *Salmonella* behaves in cells after invasion through this route. Indeed, the interaction between EGF and EGFR triggers signaling, which leads to endocytosis of the complex followed by intracellular trafficking regulated by post-translational modification, ubiquitination and phosphorylation. Furthermore, it has been shown that high or low doses of extracellular EGF induce EGF/EGFR endocytosis in non-clathrin or clathrin-coated vesicles, respectively. Non-clathrin-coated vesicles lead to EGFR degradation rather than EGFR recycling as is the case in clathrin-coated vesicle (Haglund and Dikic, 2012). Based on this well described process, we can hypothesize that depending on the saturation effect of *Salmonella* expressing Rck, bacteria could be internalized by endocytosis in a non-clathrin or clathrin-coated vesicle resulting in different outcomes (Figure 4). Moreover, it has been described that *L. monocytogenes, Escherichia coli, Staphylococcus aureus* and earlier *Shigella flexneri* enter host cells using clathrin-dependent mechanism (Clerc and Sansonetti, 1989; Veiga et al., 2007) instead of clathrin-independent mechanism for *Salmonella* in a T3SS-1-dependent invasion cell model (Veiga et al., 2007). Non-clathrin and clathrin vesicles are a weak source of membrane and extracellular material compared to T3SS-1-dependent vacuoles that fuse with macropinosomes providing a large source of membrane and extracellular fluid and thus nutrients. This process has been imaged during *Shigella*-mediated vacuole formation (Garcia-del Portillo and Finlay, 1994; Weiner et al., 2016). Consequently, non-clathrin and clathrin vesicles could represent a less favorable environment leading to bacterial destruction or *Salmonella* exit into the eukaryotic cytosol. In the cytosol, *Salmonella* may hyper-replicate dependently of T3SS-1 expression (Knodler et al., 2014). Another hypothesis would be that the vesicles could fuse with endosomes, allowing the SCV to mature. In this case, complete maturation, including SCV transport along microtubules to reach a perinuclear position and SIF formation requires the coordination of T3SS-1 and T3SS-2 effectors as mentioned above. Regarding T3SS-1 and T3SS-2 effector expression, two situations are possible: (i) the effectors of T3SS-1 and -2 have a joint action, leading to classical intravacuolar multiplication and SIF formation; (ii) their expression is unbalanced, allowing intravacuolar multiplication without SIF formation. Currently, *Salmonella* behaviors after Rck dependent internalization are under investigation in our laboratory.

## From the receptor to *Salmonella* pathogenesis

While the involvement of Rck/EGFR interaction in *Salmonella* internalization is clear and well characterized *in vitro*, the importance of Rck/EGFR interaction in *Salmonella* pathogenicity is poorly understood. An *in vivo* study was performed with *S*. Typhimurium in a mouse model of intestinal persistence (an asymptomatic carrier state model) and showed that Rck plays a role in the intestine (Dyszel et al., 2010). This is consistent with the fact that SdiA, the quorum sensing regulator of *Salmonella*, is a key element in triggering Rck expression (Ahmer et al., 1998). Indeed, the deletion of *rck* in *S*. Typhimurium results in a decrease in the bacterial fitness phenotype, compared to a strain expressing Rck. This was observed from 1 to 3 weeks post-infection (Dyszel et al., 2010). Taken together, these data suggest that Rck plays an intestinal role during *Salmonella* infection at least in mice. It would be interesting to study the role of Rck in the asymptomatic carrier state of *S*. Typhimurium in chickens as it is of high importance for human contamination (Duchet-Suchaux et al., 1997; Chaussé et al., 2011; Boumart et al., 2012). This model of asymptomatic carrier state is interesting as studies show that the T3SS-1 is not essential in the chicken asymptomatic carrier state model (Jones et al., 2007; Desin et al., 2009).

Studies by Dyszel et al. and Wiedemann et al. lead to several hypotheses concerning the role of Rck and EGFR interaction in *Salmonella* intestinal colonization (Dyszel et al., 2010; Wiedemann et al., 2016). This interaction results in *Salmonella* internalization (Wiedemann et al., 2016). It has been reported that EGFR is expressed by the epithelium of the intestine (Booth and Potten, 1996), but it is restricted to the basolateral surface of polarized epithelial cells (Singh and Coffey, 2014). As *S*. Typhimurium can invade polarized cells through the apical and basolateral sides (Criss and Casanova, 2003), we thus hypothesize that immediately on *Salmonella* crossing the intestinal barrier, the Rck/EGFR could interact, allowing enterocyte invasion through the basolateral side. This reinfection could increase the intestinal colonization and perhaps persistence in the intestine. Another non-exclusive hypothesis is based on the observation that *Salmonella* is able to use its T3SS-1 to disrupt the intestinal barrier function to facilitate bacterial invasion and colonization (Zhang et al., 2013). Thus alternatively, intestinal invasion by *Salmonella* at the apical side may induce a redistribution of the EGFR localization, promoting Rck/EGFR interaction at the apical side, resulting in an increase in *Salmonella* internalization and colonization of the intestine. Further studies are required to decipher the role of Rck/EGFR interaction during *Salmonella* intestinal colonization. Another possibility is that the role of Rck-mediated invasion is not limited to the intestine. Considering that *Salmonella* is able to disseminate and colonize deep organs including the liver (Vazquez-Torres et al., 1999), which expresses a high level of EGFR protein on hepatocyte cell surfaces, Rck/EGFR interaction could be implicated in *Salmonella* hepatocyte invasion, leading to liver colonization. In addition, the fact that Rck induces *Salmonella* resistance to complement killing suggests a role of Rck in the systemic function. The challenge now is to experimentally test these different hypotheses.

## Conclusion

To infect a host successfully, *Salmonella* has to cross several barriers and multiply in different tissues during the course of infection, despite a broad spectrum of defense mechanisms developed by the host. Invasion of non-phagocytic cells and colonization are thus critical for *Salmonella* pathogenesis. To achieve this, *Salmonella* has to coordinate the expression of virulence entry genes with their function. Although the conditions of Rck expression and its implication in pathogenesis have not been completely elucidated, the role of Rck in *Salmonella* internalization following its interaction with EGFR has been clearly demonstrated. As EGFR activation regulates several cell processes such as cell proliferation-differentiation, survival, transformation and migration, the interaction of Rck with EGFR may also have other impacts on host cell functions, presumably to the benefit of *Salmonella*. Major challenges of future research will be to determine how *Salmonella* coordinates the expression of the various bacterial entry factors with their activities during the different stages of host infection. Currently, we know neither the circumstances leading to the use of these different entry factors, nor the hosts in which they are involved, considering the fact that *Salmonella* can infect plants and warm-blooded animals.

Finally, according to the pathobiome concept, it is important to take into account the fact that the innate immune responses and host microbiota mainly constituted of commensal bacteria and residing on the mucosal surface play a critical role in the control of bacterial colonization (Stecher and Hardt, 2008; Vayssier-Taussat et al., 2014; Ribet and Cossart, 2015). Indeed, during *Salmonella* infection, the bacterium is able to use the host response to over-compete with the microbiota composition. More particularly, *Salmonella* invasion of the intestinal epithelium causes gut inflammation, which alters the composition of intestinal microbiota. *Salmonella* growth is thereby advantaged in this environment, enhancing *Salmonella* intestinal colonization (Stecher et al., 2007; Winter et al., 2010). Moreover, the physiological aspects of metabolism utilized by the bacteria when growing in diverse and varied environments are now considered as an important part of the virulence mechanisms that can influence the invasion of *Salmonella*.

## Author contributions

AW outlined the manuscript. JM, IV-P, SH, and AW wrote the manuscript. JM, SH and OG prepared the figures. JM, IV-P, SH, OG, PV, and AW revised the manuscript critically. All authors read and approved the final version of the article.

### Conflict of interest statement

The authors declare that the research was conducted in the absence of any commercial or financial relationships that could be construed as a potential conflict of interest.

## Abbreviations

AHL: Acyl Homoserine Lactone
Dsb: Disulfide bond
EGF: Epidermal Growth Factor
EGFR: Epidermal Growth Factor receptor
OMP: Outer Membrane Protein
PagN: PhoP activated gene N
PH: Pleckstrin Homology
Rck: Resistance to complement killing
SCV: *Salmonella* Containing Vacuole
SdiA: Suppressor of cell division inhibition A
SH2: Src Homology 2
SIF: *Salmonella* Induced Filament
SPI: *Salmonella* Pathogenicity Island
T3SS: Type III Secretion System.
